# ADVANCED ILLNESS AMONG ELDERLY NURSING HOME RESIDENTS WITH ALZHEIMER’S DISEASE AND RELATED DEMENTIA

**DOI:** 10.1093/geroni/igz038.1882

**Published:** 2019-11-08

**Authors:** Tadeja Gracner, Mark Sorbero, Patricia W Stone, Mansi Agarwal, Andrew W Dick

**Affiliations:** 1 RAND Corporation, Washington, District of Columbia, United States; 2 RAND Corporation, Pittsburgh, Pennsylvania, United States; 3 Columbia University School of Nursing, New York, New York, United States; 4 RAND Corporation, Boston, Massachusetts, United States

## Abstract

Alzheimer’s disease and related dementia (AD/ADRD) are leading causes of mortality in the United States. Identifying advanced illness (AI) in NH residents is key for developing therapeutic and palliative care plans for end of life. We refined and extended existing measures of AI in NH residents with AD/ADRD and described patterns of survival for each measure. Using the Minimum Data Set (MDS; 2011 to 2013) linked to vital status (through 2016), we defined categories of AD/ADRD residents at AI onset: (1) those with ADRD, (2) and those with both, AD and ADRD. We estimated survival functions and multivariable duration models to describe patterns of survival from AI onset until death, stratified by AD/ADRD classifications, sex and functional status at AI onset, conditional on socio-demographics and co-morbidities. We limited our sample to adults ages >64 for whom we observed the incident AI assessment in the MDS. Median survival was 229 days for all classifications of AI, but higher for those with only ADRD (300 days). Survival declined substantially for residents with eating difficulties; to 122 days for residents with AD and ADRD. A stark survival decline (40 days) occurred among residents with shortness of breath. Across all AI classifications, survival was negatively associated with male sex, age, diabetes, substantial weight-loss and events such as heart failure. Depression, hypertension, and UTI were associated with small or insignificant increases in mortality risk. AI can be defined using MDS data, allowing for examination of policies designed to improve end of life care.

